# Infectious Diseases and the Lymphoid Extracellular Matrix Remodeling: A Focus on Conduit System

**DOI:** 10.3390/cells9030725

**Published:** 2020-03-16

**Authors:** Fernanda N. Morgado, Aurea Virgínia A. da Silva, Renato Porrozzi

**Affiliations:** Laboratório de Pesquisa em Leishmaniose, Instituto Oswaldo Cruz, Fiocruz, Rio de Janeiro-RJ 21040-360, Brazil; aurea.silva@ioc.fiocruz.br

**Keywords:** extracellular matrix, conduit system, remodeling, infectious diseases, spleen, lymph node

## Abstract

The conduit system was described in lymphoid organs as a tubular and reticular set of structures compounded by collagen, laminin, perlecan, and heparin sulfate proteoglycan wrapped by reticular fibroblasts. This tubular system is capable of rapidly transport small molecules such as viruses, antigens, chemokines, cytokines, and immunoglobulins through lymphoid organs. This structure plays an important role in guiding the cells to their particular niches, therefore participating in cell cooperation, antigen presentation, and cellular activation. The remodeling of conduits has been described in chronic inflammation and infectious diseases to improve the transport of antigens to specific T and B cells in lymphoid tissue. However, malnutrition and infectious agents may induce extracellular matrix remodeling directly or indirectly, leading to the microarchitecture disorganization of secondary lymphoid organs and their conduit system. In this process, the fibers and cells that compound the conduit system may also be altered, which affects the development of a specific immune response. This review aims to discuss the extracellular matrix remodeling during infectious diseases with an emphasis on the alterations of molecules from the conduit system, which damages the cellular and molecular transit in secondary lymphoid organs compromising the immune response.

## 1. Introduction

Extracellular matrix (ECM) comprehends a meshwork of macromolecules such as fibrillar proteins, glycoproteins, enzymes, and proteoglycans, among others. It gives structural and functional support to the cells. ECM is responsible for the development and maintenance of functional activities of organs and allows cellular migration and activation. In secondary lymphoid organs, dendritic cells (DCs), T and B lymphocytes are disposed in a highly organized manner in order to generate an immune response to antigens (Ags). T and B cells are segregated in specific areas, and through the arrangement of ECM, the cells move, interact, and respond to the arrival of Ags. In these organs, in the core of the reticular network, three-dimensional structures of collagen wrapped by fibroblast reticular cells are formed. These 3D structures have been called conduit systems and are in charge of carrying small molecules such as cytokines, chemokines, Ags, and immunoglobulins. Conduits are essential to efficiently transport molecules through the parenchyma to specific regions where they are needed. In this review, the structure and function of ECM and the conduit system of the lymph nodes and spleen are presented, and the consequences of infection-causing remodeling of ECM compounds are addressed. 

## 2. Extracellular Matrix Composition in Lymph Node and Spleen

In the multicellular organism, not only are cells responsible for organ development during the embryonic period but also the extracellular matrix and their components, indispensable to provide the cell stimulus, improve the cell structural scaffold and biochemical mechanism [1,2,3,4]. In the lymphoid organs, the extracellular matrix components such as collagens and glycoproteins are produced by mesenchymal cells, mainly fibroblasts [5]. In lymphoid organs, the extracellular matrix is crucial to create a microenvironment that favors the immune response development [6,7]. The intracellular microenvironment can interact with the extracellular matrix through the binding of integrin in cell transmembrane with extracellular matrix molecules such as laminin and fibronectin, that interact with other extracellular matrix proteins [8,9,10,11,12]. The interaction between cells and the extracellular matrix influences cell migration, communication, adhesion, and proliferation.

The immune response is mediated by numerous cells, such as phagocytes, mastocytes, natural killer cells, dendritic cells, granulocytes, and lymphocytes, which show activity in multiple locations of the organism [13]. The lymph node is one example of a lymphoid organ that receives immune cells from the bone marrow and shelters these cells inside the specific niches like lymphoid follicles rich in B lymphocytes and medullary zone, rich in T lymphocytes, macrophages, and many other cells of the immune system [14]. The lymph nodes have three compartments: The cortex, paracortex, and medullary area. The lymphocytes enter lymph nodes through the high endothelial venules (HEVs), achieving the paracortex [11,14,15]. The principal extracellular matrix components of the lymph node are collagens, glycoproteins, and proteoglycans [16].

The role of the extracellular matrix in the lymph node is the structural scaffold of T and B niches, the maintenance of morphological integrity, regulation of cell growth, cell division, cell migration, and the distribution and accumulation of chemokines, cytokines, and growth factors [15,16,17,18]. This organ has a specific role in the body complex. The filter system is divided into two levels: The subcapsular sinus level and the interface between the sinus and parenchyma [19]. The lymph and the antigens enter through efferent lymphatics, reaching subcapsular sinus and selectively cross the lymph nodes tissue.

A reticular network forms the extracellular matrix of lymph nodes. It is composed by types I, III, and IV collagens, laminin, elastin, tenascin, entactin, vitronectin, and fibronectin [14,15,19,20,21]. The cortex stroma is also formed by a network of collagen fibers enwrapped by the reticular fibroblast, forming a transport system of low weight molecules called the conduit system [15,19,20,22,23].

One of the roles of the extracellular matrix in the lymph node is to maintain specialized compartments. Another function is to improve the strength and elasticity given by collagen and elastin fibers. Adhesion and migration roles are given by integrins and reticular fibroblasts of the lymph node scaffold [22]. The migration of the lymphocytes and other cells is not exclusively made by integrins and integrins receptors but through the chemokines gradient and by an interaction between fibroblastic reticular and migrant cells. This migration process is selected by the size and by the biochemical factors [15,19,24,25,26].

The spleen is another secondary lymphoid organ that receives immune cells produced by hematopoiesis and lymphopoiesis, and T lymphocytes from thymus after maturation stage [27]. Analogous to lymph nodes, the spleen is segregated in T and B cell areas. Although segregated, these regions are adjacent, which enables cell cooperation. The spleen is organized in two main regions: (1) the white pulp, where T cells are concentrated in lymphatic periarteriolar sheath, whereas B cells are concentrated in lymphoid follicles and (2) the red pulp, where different resident or migratory cells (lymphocytes, macrophages, fibroblasts, and dendritic cells) are observed [28,29]. The extracellular matrix is important to maintain this splenic compartmentalization and shows the different composition and structural characteristics depending on the region, influencing the cell type migrating to specific areas. Splenocytes enter the spleen through sinusoids in the red pulp [24,29]. There, the cells interact with a splenic extracellular matrix composed by proteins of an interstitial matrix such as types I, II, III, V, and XI collagen, fibronectin, and tenascin C that interact with other extracellular matrix components such as laminin, collagen IV and heparan sulfate proteoglycans [30]. This interaction between the cell, collagen, proteoglycans, and glycosaminoglycans promotes the connection and communication of the cell with the extracellular matrix. Interestingly, the extracellular matrix of the spleen is a loose connective tissue composed of apart from structural proteins glycosaminoglycans such as chondroitin, dermatan and keratin sulfate, and some glycoproteins such as tenascin-c and vitronectin. These interactions confer to the spleen the flexibility and tensile strength [29,30,31,32].

## 3. Structure and Function of the Conduits System in Secondary Lymphoid Organs

In secondary lymphoid organs, principally lymph nodes and spleen, the organization of specific areas housing T or B cells, macrophages, and dendritic cells are crucial to cell-cell interaction in to establish the adaptative immune response. The ECM plays a fundamental structural and functional role in the distribution of the different cell types, facilitating cell activation, proliferation, migration, and effector function, as described above.

**Figure 1:**
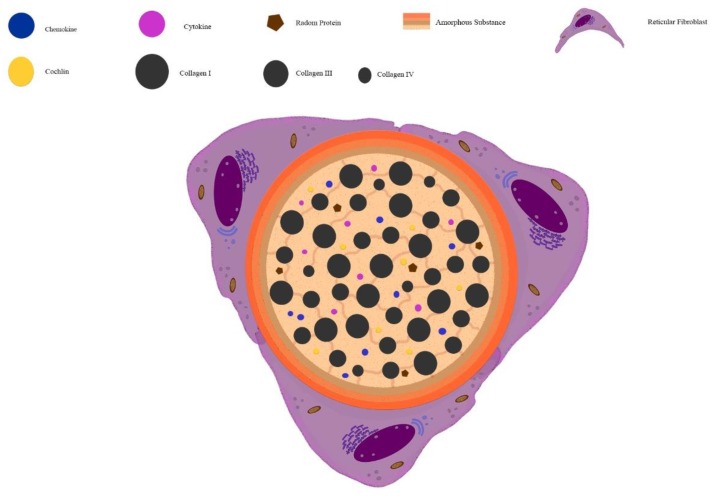
Illustrative scheme of conduit channel. The conduit system is responsible for driving, selectively, proteins, chemokines, and cytokines through the interaction between proteins and proteins associated with collagen fiber into the conduit lumen. Fibrillin molecules maintain the collagen fibers bound between each other and the basal membrane. The conduit lumen is wrapped by an amorphous substance that is secreted by the fibroblast reticular cells. The amorphous substance is composed of laminin, heparan sulfate, nidogen, perlecan, fibrillin, and other proteins of basal proteins. The scheme is based on conduit transverse.

The primary adaptive immune responses initiate at the T cell areas of the secondary lymphoid organs, where naive T cells find DCs, recognize, process, and present antigens on its surface. There was speculation in 1964 about the role of the reticular fibers network of the lymph node in transporting soluble antigens from the inoculation site to the DCs [33]. In the core of the reticular network, a three-dimensional structure is formed. This structure is composed of types I and III collagen wrapped by fibroblast reticular cells (FRC) [22,34,35,36], known as the conduit system (Figure 1). Within this specialized structure, soluble low molecular weight molecules, bellow 70 kDa in T cell area [37], are transported from one area to another. It includes cytokines, chemokines, Ag [36], and more recently, it has been shown that even molecules as large as IgM [38]. Recent advances in the structure of the conduit system have demonstrated a pericellular basement membrane surrounding a cell-free lumen composed of specialized matrix compounds. This basement membrane is similar to that underlining endothelial cell, and it contains laminin-411 and laminin-511, which may serve as a barrier [39].

Additionally, type IV collagen, heparan sulfate proteoglycan, perlecan, nidogen 1, and fibronectin are also found in the basal membrane [36]. It seemed that a microfibrillar zone exists connecting the basal membrane and the collagen core. Fibrillin 1 and 2 are arranged as the backbone of fibrillar aggregates of 10–12 nm in diameter, named microfibrils [40,41], and might play a role in the stability and elasticity of the system.

The distribution of conduits has been described in the lymph nodes and occurs differently, depending on the located region. The conduit network is denser and more branched in the T cell zones than in the B cell follicle. These differences are determined during the lymph nodes development [25]. In neonates, there are no follicles of B cells but a dense conduit network observed in developed T cell zones [25]. During lymph node development, new B cells enter the organ leading to an intense remodeling of the follicular conduit network but maintaining the connectivity of the conduit system [25]. The B cell follicles develop in the periphery of T cell zones making the conduit network sparse and enwrapped by follicular dendritic cells replacing the fibroblast reticular cells [25]. Thus, the conduit network in lymph nodes transports low weight molecules and antigens from the subcapsular sinus to deep regions to encounter follicular dendritic cells. It may be a strategy to collect antigens even in the absence of soluble antibodies and to start the immune response of B cells.

The conduit system also transports antigens to high endothelial venules (HEV) and T cell region (paracortex). On the other hand, dendritic cells originating from inflamed tissues migrate to draining lymph nodes via lymphatic vessels, enter the paracortex region, and also migrate to HEV [42]. There, DC can activate the specific T cells that get into the lymph node through HEV [42]. In the T cell zone, the conduit system is ensheathed by FRCs, which helps ensure an appropriate seal around the lymph transporting conduit system and mature DCs move through lymphocytes independently of the conduit system [36].

Beyond antigen transportation, the conduit network plays a role in the migration of DC, B, and T cells, conducting these cells to their segregated areas but enabling them to interact and cooperate [24]. Chemokines are transported through this system, also addressing these cells [37].

Different from lymph nodes, which receive Ags directly from the afferent lymphatic vessel, most Ags gain the spleen by the blood vessel. The blood enters the spleen directly in the red pulp, and a minor part of it reaches the marginal zones located between the white and red pulp. The white pulp is restricted to the lymphocytes, and soluble Ags, and other cells cannot reach this area [29]. However, it has been shown in the presence of the conduit system in this organ [43]. These authors observed in B cell follicles, the B cell associated with the conduit without expression of ER-TR7^+^, which shows a peculiar heterogenicity of the FRC. Chemokines and antigens are also transported by the conduits, allowing the generation of immune responses.

## 4. Extracellular Matrix and Conduit System Remodeling in Chronic and Infectious Diseases

Secondary lymphoid organs maintain active homeostasis in a steady state. Generally, they are organized as T and B lymphocytes specific areas. These specific compartments are adjacent, allowing cell-to-cell cooperation and activation of the immune response. The adaptive immune response starts at T cell zones. They are composed of a specialized extracellular matrix and the conduit system rich in fibroblast reticular cells. In this area, chemokines and cytokines are expressed by FRC and dendritic cells. They are transported through conduits, which address lymphocytes to specific areas maintaining the organization of compartments in secondary lymphoid organs. During infections, the lymphoid extracellular matrix may suffer dramatic remodeling, which plays a role in the development of a specific immune response. This process improves blood flows, immune cell trafficking, and angiogenesis, resulting in inflammatory reaction and organ enlargement. Thereby, extracellular matrix remodeling promotes the assembly of a pathogen-specific immune response. As an example, lymph nodes are constructed by an intricate network of endothelial and mesenchymal stromal cells. These change their composition after herpes simplex virus type-1 (HSV-1) infection [44]. In this case, the recruitment of lymphocytes to lymph nodes induces the increasing of stromal cell numbers (Lymphoid Stromal Cells - LSC, fibroblast reticular cells, lymphatic endothelial cells, and blood endothelial cells) [44]. The number of fibroblast reticular cells in the inflamed lymph nodes increases as a response to infection, persisting for more than three months to return to the steady-state after pathogen clearance [44]. Most of the proliferation and gene regulation of LSCs occur in the first seven days after infection. After that, they contract gradually [44]. The transcriptional changes result in cell division, antigen presentation, extracellular matrix, apoptosis, and immune response. The observed changes appear to be induced by IFN-α signaling [44]. Because of the activation and growth of FRC, T and B lymphocytes also increase with the lymph node enlargement, and B cell zones remained enlarged for 30 days after infection [44,45]. FRC and lymphatic endothelial cells up-regulate interleukin-7 (IL-7) expression responding to viral infection contributing to lymphocyte survival, remodeling, and reconstruction of the distinct lymph node microenvironment [46].

The extracellular matrix is an active participant in the development of immune response upon infection. Type VII collagen, a compound of extracellular matrix in the skin, the conduits in lymph nodes, and spleen, may capture cochlin from the lumen of the conduit systems [47]. Cochlin is an ECM protein produced by follicular dendritic cells in B cell follicles conduits and plays a role as an innate immune activator [48]. During infectious diseases, cochlin is processed by aggrecanase, releasing LCCL domain that activates macrophages and neutrophils [47]. The importance of the collagen VII-cochlin axis during bacterial infections was evidenced by the reduced IL-6 and IL-1β expression and by the increased bacterial colonization when collagen VII is genetically lost [47]. It had also been demonstrated by the reduced survival of cochlin knockout mice infected with *Pseudomonas aeruginosa* and *Staphylococcus aureus* [48].

**Figure 2:**
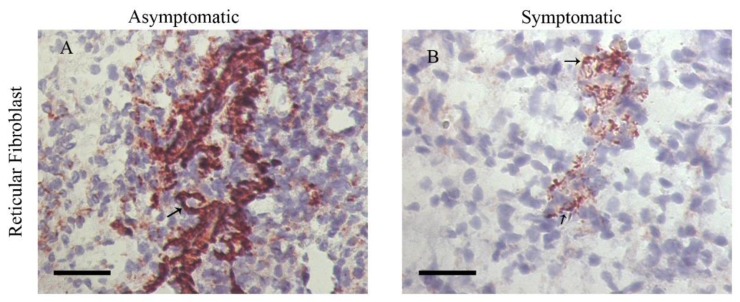
Detection of fibroblast reticular cells in the spleen of chronically infected dogs with Leishmania infantum by immunohistochemistry. (**A**) Infected and asymptomatic dog. (**B**) Infected and symptomatic dog. In this figure, the spleen of the symptomatic dog shows fewer fibroblast reticular cells than the asymptomatic dog. Fibroblast reticular cells are represented in red color in the figure.

**Figure 3:**
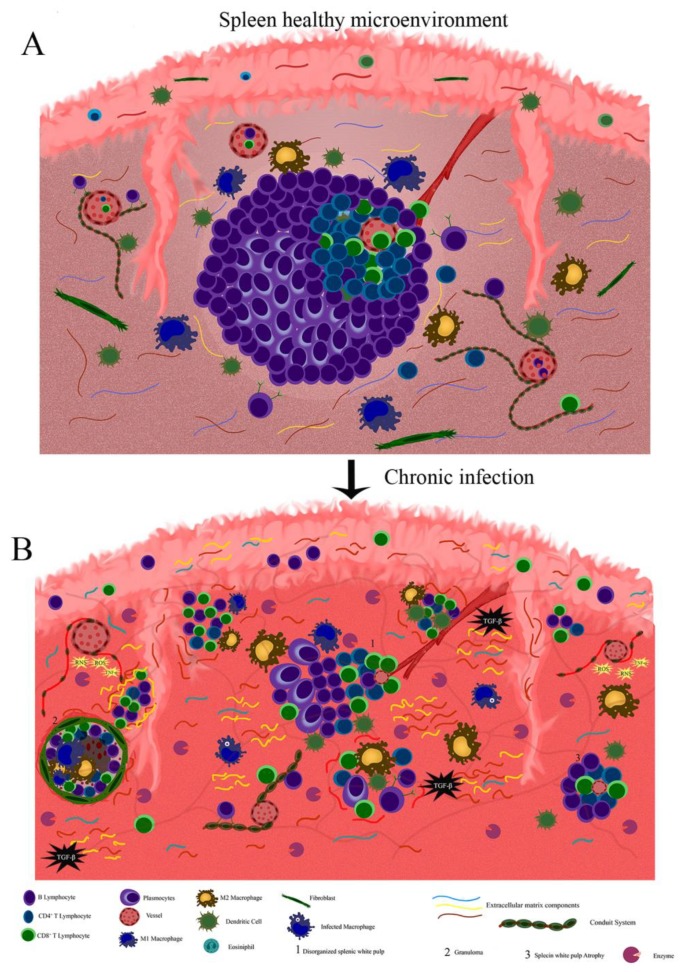
Illustrative scheme of the splenic microenvironment. (**A**) Organized spleen: In this illustration, it is possible to visualize an organized splenic with pulp and splenic compartmentalization such as germinal center and lymphatic periarteriolar sheath, marginal zone and capsule to exert its physiological role. As represented, the extracellular matrix maintains its normal composition. (**B**) Disorganized spleen: Illustrative scheme of a chronically inflamed microenvironment of spleen tissue. During chronic inflammation, the extracellular matrix undergoes remodeling favoring the entry of new inflammatory cells. In persistent inflammation, the extracellular matrix loses its remodeling control showing an increase of cytokines such as Tumour Necrosis Factor-α (TNF-α) and Transforming Growth Factor-β (TGF-β) together with high production of matrix metallopeptidases and microbicidal molecules such as Reactive Nitrogen Species (RNS), Reactive Oxygen Species (ROS), and lysosomal enzymes. These events lead to tissue damage by breaking or accumulating some structural proteins like laminin and collagen. In this situation, a cell reduction and the white pulp atrophy are commonly observed.

After pathogen clearance, the inflammatory reaction reduces, and lymphoid organs return to normal size and steady-state. In chronic infections, the persistence of pathogens and antigenic stimulus lead to permanent remodeling that triggers tissue damage. Gradually, the inflammatory reaction is hereby replaced by fibrosis, and in some situations, the changes in the extracellular matrix composition may be irreversible or may alter the function of the organs. This effect has been described in a variety of chronic and infectious diseases. In HIV, the changes in the lymphoid tissue microenvironment are accompanied by fat or fibrosis deposition. They may also be attributed to a loss of leukocytes’ communication and the surrounding stromal cells [49]. These cells produce the extracellular matrix components and the growth factors necessary for cell migration, cell proliferation, and lymphoid tissue function [49]. In canine leishmaniasis, the persistence of *Leishmania* amastigotes induces a chronic inflammatory reaction that ends in a spleen and lymph node fibrosis/collagen deposition [50,51]. Laminin and metallopeptidase-9 are also increased in the spleen of dogs with an advanced infection, suggesting an intense process of extracellular matrix remodeling [50]. In the canine leishmaniasis model, these alterations in the splenic extracellular matrix have been associated to a reduced CXCL13 expression, reduced fibroblast reticular cells (Figure 2), CD4 cells, lymphatic periarteriolar sheath atrophy, lymphoid follicle atrophy, and germinal center disruption (Figure 3) [50,51,52,53].

Some pathogens have developed the ability to use the mechanisms involved in extracellular matrix remodeling to persist and to disseminate inside the host. For example, HIV interacts with fibronectin, one of the components of the extracellular matrix, which facilitates CD4 T lymphocytes infection in vivo [54]. The binding of gp120 envelope protein mediates this interaction with the extracellular matrix to the III1-C region of fibronectin [54]. In the spleen of a chicken model, genotype VI Newcastle disease virus promotes metalloproteinase (MMP)-13 and -14 upregulation and consequent extracellular matrix degradation through collagen breakdown [55]. The authors suggested that, as the extracellular matrix components interfere with viral spread, extracellular matrix degradation facilitates viral spread, resulting in higher viral load [55]. Collagen destruction was also demonstrated in the spleen of chicken infected with infectious bursal disease virus [56]. Collagen degradation begins three days post-infection in the antigen-trapping zone and impairs tissue organization contributing to permanent immunosuppression [56].

The conduit system also changes during infectious diseases. The fibroblast reticular cell, the main cell that covers the conduit system may be a target during infectious diseases. For instance, in HIV and Simian Immunodeficiency Virus (SIV) infection, the lymph node FRC network is replaced by fibrosis (collagen deposition), impairing the production of IL-7, leading to T cell depletion and immunosuppression [57]. In the mouse model of persistent infection by lymphocytic choriomeningitis virus (LCMV), the FRC network was infected and altered by the action of CD8 T cells [58]. Interestingly, Programmed death – ligand 1 (PD-L1) was up-regulated on FRC, reducing the activation of CD8 T cells and, consequently, the immunopathogenesis, thereby contributing to viral persistence [58].

Depending on the model of study and, consequently, the course of infection, the effects on the FRC may vary. For example, the experimental infection of the murine model with *Leishmania infantum* leads to an increase in FRC [59]. Upon infection, polynutrient-deficient mice showed a reduction in FRC, dendritic cells, and macrophages when compared with well-nourished mice [59]. *Leishmania* was co-localized with dendritic cells and high endothelial venules associated with an intact conduit network [59]. In infected and malnourished mice, the authors observed an early parasite visceralization when compared to well-nourished and infected mice. They suggested that early visceralization of amastigotes was not due to a passive movement through a leaking barrier, but to a reduced number of lymph node phagocytes [59]. They further suggested a role of conduit system flow in the early visceralization of *Leishmania donovani* [59].

Lymphotoxin beta (Ltb) plays a role in splenic architecture, developing conduits along the marginal zone and recruiting CD169+ macrophages [60,61]. In the model of LCMV infection, extracellular distribution of virus along the splenic conduits is necessary for inducing systemic levels of IFN-I and is dependent on the presence of Lymphotoxin B-induced conduits [62]. In the presence of IFN-I, cellular exhaustion is induced through PD-L1 and IL-10 expression inhibiting the response of virus-specific CD8+ T cells and favoring virus persistence [63,64].

Recently, Reynoso et al. [65] demonstrated that vaccinia virus and zika virus are transported through conduits in lymph nodes to have access to cells in order to infect them. They evidenced these viruses rapidly infecting the cells, which were adjacent to conduits [65]. Prions also may traffic through lymph node conduits [66]. Moreover, in a study of the role of perinodal adipose tissue (PAT) during immune responses, the authors observed the fluid of PAT enter the lymph node through PAT-LN conduits contributing to the immune response [67]. Besides, *Staphylococcus aureus* intradermally or intravenously infected may use PAT-LN conduits to infect PAT [67].

A notable example of lymphoid extracellular matrix remodeling is the formation of tertiary lymphoid tissue during chronic inflammation in various non-lymphoid organs. These structures are able to maintain a cellular organization similar to B and T cell areas of secondary lymphoid organs such as lymph nodes [68]. The maintenance of tertiary lymphoid tissue is dependent on lymphtoxin β that can be expressed by B lymphocytes [68,69]. Tertiary lymphoid tissue is composed of a variety of hematopoietic cells, high endothelial venules, and follicular dendritic cells [69]. An intricate network formed by FRC is also observed, and these cells play an important role in the induction and persistence of tertiary lymphoid tissue since they produce CCL21, express lymphtoxin β receptor and form a functional conduit system [69]. As observed for lymph nodes, conduits are dense in T cell areas and sparser in B cell areas from tertiary lymphoid tissue [68].

## 5. Final Considerations

**Figure 4:**
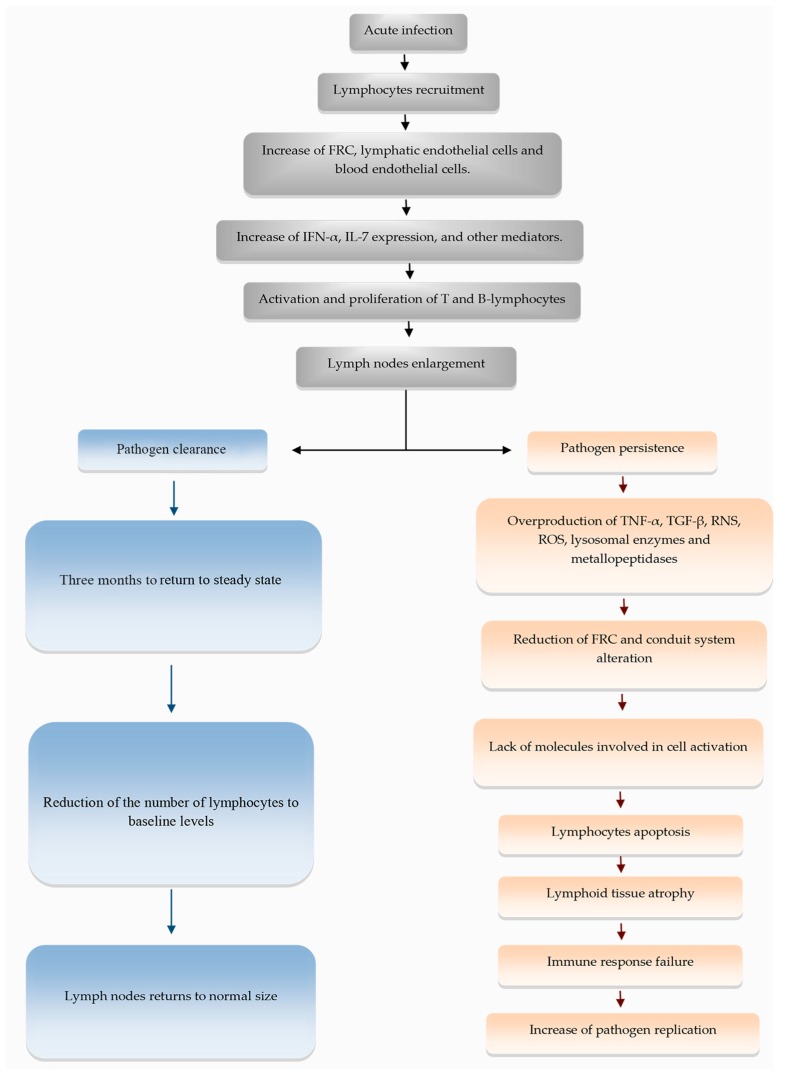
Flowchart showing briefly the steps of extracellular matrix remodeling and changes in lymphoid tissue during acute and chronic infectious processes.

The remodeling process of the extracellular matrix of secondary lymphoid organs plays a vital role during immune responses against infectious agents and has been studied in a variety of models. However, little has been described about the impact of infections on the conduit system. Despite scarce data observed and discussed in this paper, we can conclude that the processes of infection by different etiological agents generate changes in the cellular and fiber components of conduits. In some situations, parasites may use the conduit system or induce changes in it to favor the spread of pathogens and their permanence in the host. During acute through chronic infection, several events aiming to control the microbial spreading result in extracellular matrix remodeling and/or conduit system disruption. For example, after microbial infection the increase of cytokines such as TNF-α and TGF-β together with a high production of matrix metallopeptidases and microbicidal molecules, such as RNS, ROS, and lysosomal enzymes, lead to the tissue damage, breaking or accumulating matrix molecules (Figure 4). Thus, to avoid such events, it is crucial to maintain an appropriate cooperation and activation of the immune system in secondary lymphoid organs. One question that arises is concerning if killing the microorganism using drugs will further restore the architecture of the organ. Since chronic infection causes intense disorganization and fibrosis, it seems that preventing or limiting such events may be the best way to restore homeostasis. The application of coadjuvant drugs, such as pentoxyfylline [70] and infliximab [71], may protect the tissue from a high production of TNF-α, which is also responsible for the activation of several enzymes, limiting the matrix disorganization. Unfortunately, several other molecules contribute to tissue damage, and further research is needed.
